# Impact of incision type in breast cancer-conserving mastectomy: a comparative analysis of outcome

**DOI:** 10.1007/s13304-025-02443-3

**Published:** 2025-10-31

**Authors:** Gianluca Vanni, Marco Pellicciaro, Marco Materazzo, Alice Bertolo, Amir Sadri, Alessio Fazi, Benedetto Longo, Massimiliano Berretta, Valerio Cervelli, Oreste Claudio Buonomo

**Affiliations:** 1 Breast Unit Policlinico Tor Vergata, Department of Surgical Science, Tor Vergata University, Viale Oxford 81, 00133 Rome, Italy; 2 Department of Surgical Science, PhD Program in Applied Medical-Surgical Sciences, Tor Vergata University, Rome, Italy; 3 Plastic Surgery, Great Ormond Hospital for Children, NHS Foundation Trust, London, WC1N 3JH UK; 4 Plastic and Reconstructive Surgery at Department of Surgical Science, Tor Vergata University, Viale Oxford 81, 00133 Rome, Italy; 5 Department of Clinical and Experimental Medicine, University of Messina, 98100 Messina, Italy; 6 University of Basilicata, Via Nazario Sauro, 85, 85100 Potenza, Italy

**Keywords:** Conserving mastectomy incision, Breast reconstruction complications, Breast cancer surgery, Implant-based reconstruction

## Abstract

Conserving mastectomy is widely used due to its cosmetic benefits and the possibility of undergoing immediate breast reconstruction. The types of incisions vary, and the best method remains a topic of debate. In this study, we evaluate the impact of the incision type on complication outcomes. A retrospective analysis was conducted, considering all conserving mastectomies and dividing them into groups based on the incision type. A total of 290 patients were enrolled: 34 (11.7%) had a radial incision, 95 (32.8%) transverse incision with skin or NAC excision, 48 (16.6%) inframammary fold incision, 39 (13,4%) omega incision, 37 (12.8%) periareolar incision, and 37 (12.8%) Wise-pattern incision. Flap ischemia, NAC necrosis, and skin necrosis had a higher incidence rate in patients who underwent the Wise-pattern incision and periareolar incision, with relative *p* values of <0.001, 0.041, and 0.035, respectively. Considering only nipple-sparing mastectomies in breast cancer patients, overall complications, skin necrosis, and delay in adjuvant treatments showed a higher incidence in patients who underwent Wise-pattern incisions. The relative *p* values were 0.001, 0.004, 0.054, and 0.001, respectively. In multivariate analysis, inframammary fold incision reduced the risk of nipple necrosis, while Wise-pattern incision increased the risk (OR 0.656; 3.611-fold and *p* value were, respectively, 0.026 and 0.038). Wise-pattern incision and periareolar incision increase the incidence of complications as nipple areola complex (NAC) and skin necrosis but do not affect reconstruction failure. Inframammary fold incision reduces the risk of complications. The incision should be customized based on the tumor location, biology and extent, breast volume, ptosis, type of reconstruction, and risk factors.

## Introduction

In recent decades, with the advent of breast-conserving surgery and implementation of neoadjuvant treatments, we have observed a marked reduction in breast cancer patients undergoing mastectomy [1]. Despite this significant reduction, nowadays approximately 30% of women with a breast cancer diagnosis still undergo mastectomy [2]. During the years, there have been several changes in the field of mastectomy [3]. The current mastectomy technique, when oncologically feasible, preserves the nipple areola complex (NAC) and the skin as much as possible, demonstrating oncologic safety nonetheless [4, 5]. Nipple-sparing mastectomy (NSM) allows, more frequently, immediate breast reconstruction (IBR) with advantages in terms of esthetic result, quality of life, and physiological impact [4]. Based on the different meta-analysis reported in the literature, despite the acceptable overall complication rates, conserving mastectomy has a higher risk of skin or NAC necrosis [5–7]NAC or mastectomy skin flap (MSF) necrosis are considered the most problematic complications, leading to adverse outcomes such as delayed wound healing, breast or nipple deformity, hypopigmentation in the areola complex, loss of the NAC, implant infection or loss.

Several studies have identified the risk factors of NAC or skin necrosis in patients subjected to NSM [8–10]. Some of these, such as smoking, type II diabetes mellitus, and cardiovascular system disorders, are patient-related risk factors others are correlated to surgical procedure [11]. Injury to the blood supply during the glandular and ductal tissue resection, and reduced thickness of the MSF can both increase the necrosis risk [5, 12]. Also, the type of surgical incision has been identified as a risk factor for NAC necrosis, with the highest necrosis rates in periareolar and Wise-pattern incision [9, 10, 12–14]. Various methods are used for the incision, depending on the size of the breast, tumor localization, and surgeon preference, because there are currently no guidelines on the incision method. The aim of this study is to compare the complications of conserving mastectomy followed by IBR among the different incision types and analyze the patient characteristics, tumor characteristics, and surgical factors to investigate the risk factors of complications.

## Materials and methods

**Fig. 1:**
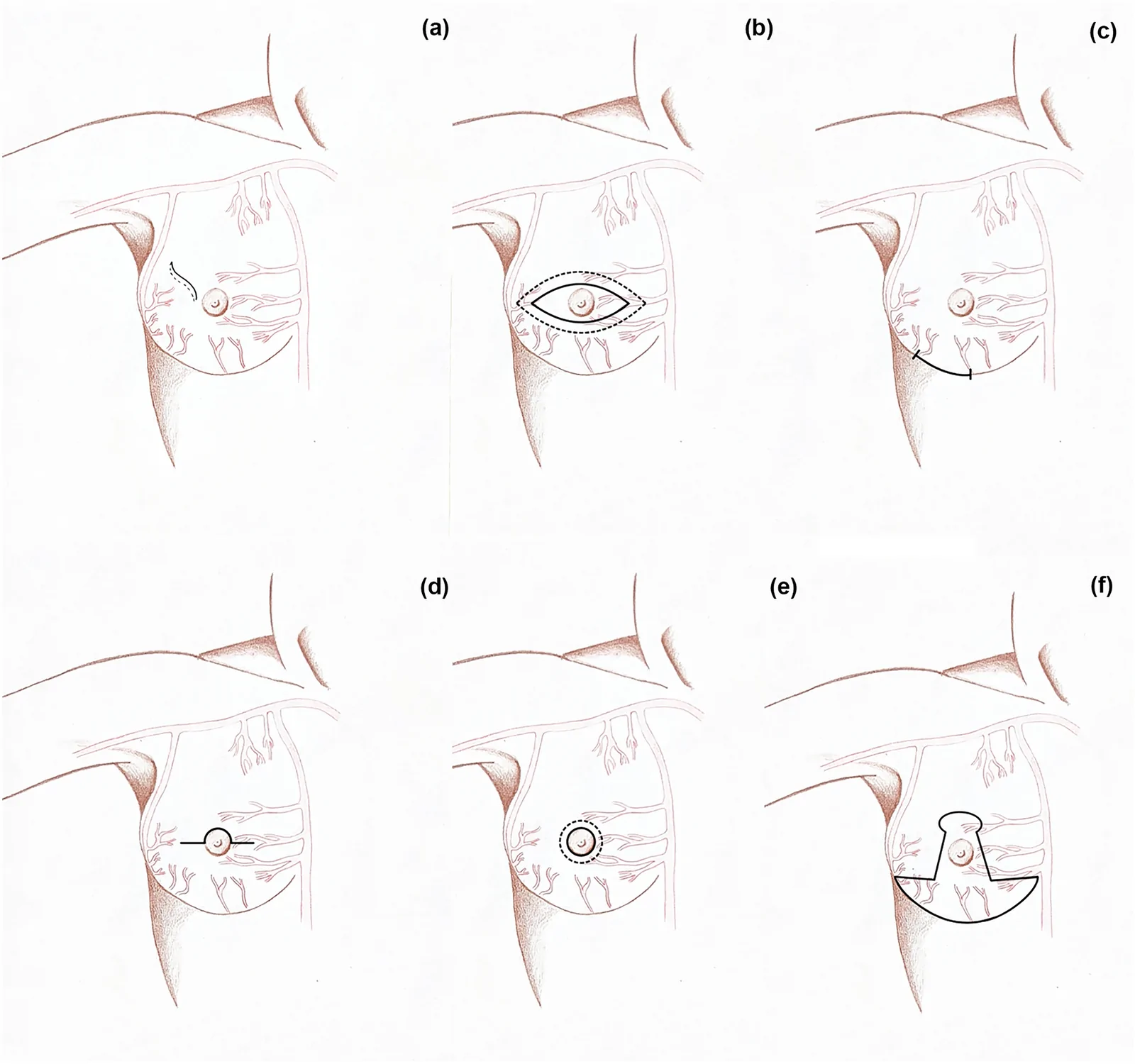
Type of incision for conserving mastectomy. (**a**) Radial incision, (**b**) Transverse with skin or NAC excision, (**c**) Inframammary fold incision, (**d**) Omega areola incision, (**e**) Periareolar incision, and (**f**) Wise-pattern incision, used to reduce excess skin

A single-center retrospective study including all patients who underwent conserving mastectomy from January 2014 to March 2024 at the Breast Unit of the Policlinico Tor Vergata, Rome, with at least 1-year follow-up was performed. All patients subjected to simple mastectomy, breast autologous reconstruction, or who were not subjected to breast reconstruction were excluded from the analysis. Age, type II diabetes mellitus, and smoking data were retrieved from preoperative clinical notes. Previous surgery and breast irradiation are reported and analyzed. Conserving mastectomy includes all patients subjected to nipple areola complex sparing, skin-sparing or skin-reducing mastectomy. Type of mastectomy and type of incision were retrieved from surgical procedure reports. Incisions were categorized as follows: radial incision (Fig. 1a), transverse with skin or NAC excision (Fig. 1b), inframammary fold incision (Fig. 1c), omega areola incision (Fig. 1d), periareolar incision (Fig. 1e), and Wise-pattern incision due to the need for reducing the skin (Fig. 1f). Inframammary fold incision, according to our policy, was performed in the lower outer quadrant as shown in Fig. 1c. Wise-pattern incision was adopted for skin reduction; in cases of skin retraction outside of the incision mark, a modified Wise-pattern incision was used [15]. A sub-analysis was performed including only patients who underwent NAC preservation and had an indication for mastectomy due to breast cancer.

Surgery was performed by an oncoplastic breast surgeon. The choice of incision was made by the operating surgeon based on the need for skin excision due to oncologic reasons, the presence of previous scars, the necessity for skin reduction, breast ptosis, and the surgeon’s individual expertise with the incision. According to preoperative marking of the patient and induction of general anesthesia, the mastectomy was performed by meticulous dissection in the plane of the subcutaneous fascia. The avascular plane between the subcutaneous fat and the glandular tissue is dissected to preserve blood supply to the derma. For dissection, scissors were used, and monopolar diathermy was used for hemostasis. In the case of nipple-sparing mastectomy, careful dissection below the nipple was performed. Intraoperative evaluation of the retroareolar tissue was carried out using frozen section analysis to assess the feasibility of preserving the nipple areola complex, provided that oncological safety and adequate tissue thickness were ensured. The breast was dissected from the pectoralis major muscle fascia using monopolar diathermy. The type of reconstruction with breast silicone prosthesis or tissue expander was achieved from surgical notes. For DTI reconstruction, a sizer, to determine the correct volume and symmetry, was used. In case of suspicions of MSF ischemia or MSF tension with the breast sizer, a reconstruction with tissue expander was preferred according to the policy of our center. Prosthesis allocation pre-pectoral with or without mesh, sub-pectoral or dual plane was reported and analyzed. In the case of dual-plane breast reconstruction, the lower inferior pole was usually covered by mesh or dermal flap sutured with the pectoralis muscle. Surgical time was considered the time of operating room occupancy, including time for the position, anesthesiologic time, and surgical procedure. Indications for mastectomies were retrieved from clinical notes and evaluated in the analysis. Data regarding tumor characteristics and TNM staging were retrieved from pathological reports and analyzed. Follow-up information was collected and compared between groups. Adjuvant breast irradiation treatments were also reported and analyzed across the different incision groups.

Surgical complications categorized according to Clavien–Dindo classification that occurred within 1 year from surgery were reported in clinical notes, and breast-specific complications were classified as: MSF ischemia (evident soft tissue ischemia without eschar formation) skin necrosis (evident soft tissue ischemia with eschar formation), nipple areola complex necrosis, delayed wound healing (full-thickness wound separation); bleeding requiring surgery, seroma, and hematoma (accumulation of fluid or clots requiring aspiration or drainage) [16]. Implant loss is the removal of prosthesis within 1 year form implant due to surgical complications: infection, exposition and/or dislocation. The time between surgery and resolution of the surgical condition was considered the interval time to adjuvant treatment. A cut-off of 60 days was considered for delay in adjuvant treatments.

The retrospective study was registered and approved by the Ethics Committee of Lazio Area 2 (Approval number 62.25CET2 ptv). No potentially identifiable images or data are presented in this study. All procedures performed in studies involving human participants were in accordance with the ethical standards with the 1964 Helsinki declaration. Formal consent was not required due to retrospective nature of the study.

### Statistical analysis

Data were collected in a Microsoft Excel Database (Microsoft 2023, Version 16.78) and analyzed using SPSS version 23.0 (IBM Co., Armonk, NY, USA). The Chi-square test or analysis of variance and ANOVA test were used to compare the patients’ characteristics, with data reported as median value and corresponding ranges. The Chi-square test was also used to compare complication rates among the types of incisions, with data reported as absolute numbers and relative percentages. Predictive risk factors for NAC necrosis were assessed using a univariate logistic regression model. Unadjusted odds ratios (OR) and 95% confidence intervals were calculated. Factors with statistical significance (*p* < 0.1) in univariate analysis were subsequently entered into a multivariate logistic regression model. Adjusted odds ratios and 95% confidence intervals were calculated based on the multivariable logistic regression model. Values of *p* < 0.05 were considered statistically significance.

## Results

Out of 799 patients subjected to mastectomy, that were the initial sample, 290 (89.5%) patients underwent conserving mastectomy and breast reconstruction with prosthesis implant or tissue expander.

Median age was 51 years [28:84]. Out of 290 patients, 98 (33.8%) underwent bilateral mastectomy, and 97 (33.4%) were subjected to neoadjuvant chemotherapy. Type of incision was a radial incision in 34 (11.7%), a transverse incision with skin or NAC excision due to cancer retraction in 95 (32.8%), inframammary fold incision in 48 (16.6%), omega areola incision in 39 (13.4%), periareolar incision in 37 (12.8%), and Wise-pattern incision in 37 (12.8%) due to the need to reduce the skin. Type of mastectomy was skin-sparing mastectomy in 103 (35.4%), NAC-sparing mastectomy in 148 (51%), skin-reducing mastectomy in 39 (13.4%). Immediate implant breast reconstruction was performed in 231 (79.7%) cases with definitive silicone breast prosthesis and in 59 (20.3%) cases with a breast tissue expander. Type of reconstruction was 134 (46.2%) pre-pectoral, 118 (40.7%) sub-pectoral, and 38 (13.1%) dual-plane. Immediate implant breast reconstruction was performed in 47 (16.7%) with the use of mesh. Indication for mastectomy was 203 (70%) invasive breast carcinoma (ductal/lobular) with or without an in situ component, 29 (10%) due to pure ductal carcinoma in situ, 14 (4.8%) due to positive margins after conserving breast surgery, and 44 (15.2%) prophylactic mastectomies. Median hospitalization was 2 [0:7] days and the median surgical time was 201 [65:395] min. Temporary MSF ischemia was reported in 13 (4.5%) patients, delayed wound healing in 19 (6.6%), NAC necrosis in 5 (1.7%), skin necrosis in 6 (2.1%), seroma in 24 (8.3%), and implant loss 16 (5.5%).

Age was significantly different between groups *p* < 0.001, with younger patients more commonly associated with the inframammary fold incision, 41 [32–59] and older patients with the Wise-pattern incision, 59 [39–84]. The median age of other groups is summarized in Table 1. The percentage of smoker patients was significantly lower in those patients subjected to Wise-pattern incision, with a *p* value less than 0.001. Pre-pectoral breast implant reconstruction was more common in patients subjected to periareolar or omega areola incisions (*p* = 0.005). The type of reconstruction according to the surgical incision is resumed in Table 1.

**Table 1:** Preoperative and intraoperative characteristics according to type of incision

	Radial incision (*n* = 34)	Incision with skin excision (*n* = 95)	Inframammary fold incision (*n* = 48)	Omega areola incision (*n* = 39)	Periareolar incision (*n* = 37)	Wise-pattern incision (*n* = 37)	*p* value
Age (years)	49[40–64]	57[31–84]	41[32–59]	52[34–78]	50[28–72]	59[39–84]	<0.001
BMI (kg/m^2^)	24.7[20.1–33.2]	26.3[20.9–34.1]	23.8[19.5–28.7]	25.6[21.3–29.0]	24.3[22.4–30.6]	27.0[23.9–37.7]	<0.001
Smokers (yes)	2(5.8%)	12(12.6%)	4(8.3%)	1(2.6%)	2(5.4%)	2(5.4%)	<0.001
Diabetes (yes)	0(0.0%)	5(5.2%)	0(0.0%)	0(0.0%)	1(2.7%)	1(2.7%)	0.001
Bilateral (yes)	15(44.1%)	14(14.7%)	16(33.3%)	17(43.6%)	15(42.9%)	21(56.8%)	<0.001
Type of mastectomy							<0.001
Skin sparing	8(23.5%)	73(76.8%)	6(12.5%)	12(30.8%)	5(13.5%)	0(0.0%)	
NAC sparing	26(76.5%)	22(23.2%)	42 (87.5%)	27(69.2%)	32(86.5%)	0(0.0%)	
Skin reducing	0(0.0%)	0(0.0%)	0(0.0%)	0(0.0%)	0(0.0%)	37(100%)	
Type implant							<0.001
DTI	32 (94.1%)	55(57.9%)	42(87.5%)	33(84.6%)	32(86.5%)	36 (97.3%)	
Tissue expander	2(5.9%)	40(42.1%)	6(12.5%)	6(15.4%)	5(13.5%)	1(2.7%)	
Type of reconstruction							0.005
Pre-pectoral	10(29.4%)	36 (37.9%)	20(41.6%)	29(74.3%)	30(81.8%)	11(29.8%)	
Sub-pectoral	14(41.2%)	51 (53.7%)	15(31.2%)	8(20.4%)	7(18.9%)	18(48.6%)	
Dual plane	7(20.6%)	8(8.4%)	13(27.1%)	2(5.1%)	0(0.0%)	8(21.6%)	
mesh (yes)	6 (17.6%)	16 (16.8%)	8 (16.7%)	7(17.8%)	7(18.9%)	3(8.1%)	0.798
Mastectomy indications							0.001
Invasive carcinoma *	24(70.6%)	67 (70.6%)	28(58.3%)	26 (66.6%)	21(56.7%)	30(81.1%)	
Pure DCIS	2(5.9%)	12(12.6%)	4(8.3%)	8 (20.4%)	5 (13.5%)	2(5.4%)	
Other**	2(5.9%)	2(2.1%)	4(8.3%)	0(0.0%)	6(16.2%)	0(0.0%)	
Prophylactic	4(10.8%)	14 (14.7%)	12(25%)	4(10.2%)	5(13.5%)	5(13.5%)	
Retroareolar tissue biopsy positive	6(23.5%)	5(5.2%)	6(12.5%)	7(20.76%)	5(13.5%)	2(5.4%)	0.137
Hospitalization (days)	2[0–7]	2[1–6]	2[2, 3]	2[0–5]	2 [2–4]	2[0–7]	0.335
Surgical time (minutes)	208[122–307]	181[65–262]	212[141–319]	209[104–256]	210[113–296]	218[131–395]	0.067

*BMI* Body mass index, *NAC* Nipple areola complex, *DTI* Direct to implant, *DCIS* Ductal carcinoma in situ

^*^Ductal or lobular carcinoma,

^**^Mixed type, previous positive resection margin; hospitalization in days, surgical time in minutes

The indication for mastectomy was significantly different between groups, *p* < 0.001, and is reported in Table 1. Hospitalization and surgical time did not differ significantly between groups, with *p* value of 0.335 and 0.067, respectively (Table 1).

The incidence of temporary MSF ischemia, NAC necrosis, and skin necrosis was higher in patients subjected to Wise-pattern incision and periareolar incision, with *p* value of <0.001, 0.041, and 0.035, respectively, as reported in Table 2. Delayed wound healing was more frequent in patients with skin excision incisions, *p* = 0.001. No significant difference was reported in term of seroma and implant loss according to skin incision, with *p* values of 0.458 and 0.185.

**Table 2:** Postoperative complications according to type of incision

	Radial incision (*n* = 34)	Incision with skin excision (*n* = 95)	Inframammary fold incision (*n* = 48)	Omega areola incision (*n* = 39)	Periareolar incision (*n* = 37)	Wise-pattern incision (*n* = 37)	*p* value
Temporary MSF ischemia	2(5.9%)	0(0.0%)	0(0.0%)	0(0.0%)	2(5.4%)	9(24.3%)	<0.001
Delayed wound Healing	2(5.9%)	14(14.7%)	0(0.0%)	0(0.0%)	0(0.0%)	3(8.1%)	0.001
NAC necrosis	0(0.0%)	0(0.0%)	0(0.0%)	1(2.6%)	2(5.4%)	3(8.1%)	0.041
Skin necrosis	2(5.9%)	0(0.0%)	0(0.0%)	1(2.6%)	0(0.0%)	4(10.8%)	0.035
Seroma	2(5.9%)	7(7.4%)	8(16.7%)	2(5.1%)	3(8.1%)	2(5.4%)	0.458
Hematoma	1(2.9%)	0(0.0%)	0(0.0%)	1(2.6%)	1(2.7%)	1(2.7%)	0.442
Bleeding	0(0.0%)	0(0.0%)	1(2.1%)	0(0.0%)	1(2.7%)	0(0.0%)	0.567
Implant loss	2(5.9%)	6(6.3%)	0(0.0%)	0(0.0%)	2(5.4%)	3(8.1%)	0.185

*MSF* Mastectomy skin flap, *NAC* Nipple areola complex

As the Wise-pattern and periareolar incisions showed significantly higher rates of nipple or skin necrosis, we further analyzed the risk factors for nipple necrosis.

### Nipple-sparing mastectomy for breast cancer

In this secondary analysis, we included all patients who underwent nipple-sparing mastectomy (NSM), with or without concomitant skin-reducing techniques, for oncologic indications. Among the original cohort of 290 patients, 44 (15.2%) were excluded due to prophylactic indications, and an additional 104 (35.9%) were excluded because they underwent skin-preserving (non-nipple-sparing) mastectomies. Consequently, 142 patients met the eligibility criteria for this analysis, with a median age of 52 years [28–82]. Median follow-up was 47 [14–119] months. Previous breast surgery was comparable among the different incision groups, as shown in Table 3 (*p* = 0.380). In contrast, a statistically significant difference was observed in the distribution of prior breast irradiation (*p* = 0.022): one patient (4.5%) in the radial incision group, two patients (25%) in the skin excision group, one patient (3.3%) in the IMF incision group, and one patient (4.4%) in the omega areolar incision group. No cases of previous irradiation were recorded in the periareolar and Wise-pattern groups.

**Table 3:** Preoperative and intraoperative characteristics according to type of incision in patients undergoing nipple-sparing mastectomy for breast cancer

	Radial incision (*n* = 22)	Incision with skin excision (*n* = 8)	Inframammary fold incision (*n* = 30)	Omega areola incision (*n* = 23)	Periareolar incision (*n* = 27)	Wise-pattern incision (*n* = 32)	*p* value
Age (years)	49 [40–64]	59 [39–82]	42 [36–62]	52 [34–77]	50 [28–72]	58 [39–79]	<0.001
BMI (kg/m^2^)	24.9 [21.0–28.3]	23.5 [20.9–26.7]	23.4 [19.5–28.7]	25.9 [21.3–29.0]	24.2 [23.8–30.6]	27.1[23.9–37.7]	<0.001
Smokers (yes)	2(9.1%)	5(62.5%)	4(13.3%)	1(4.3%)	1(3.7%)	2 (6.3%)	<0.001
Diabetes (yes)	0(0.0%)	1(12.5%)	0(0.0%)	0(0.0%)	1(3.7%)	1 (3.1%)	0.279
Bilateral (yes)	7(31.8%)	2(25%)	10(33.3%)	10(43.5%)	8(29.6%)	18 (56.2%)	0.312
Previous surgery (yes)	6(27.3%)	4(50.0%)	5(16.6%)	5(21.7%)	6(22.2%)	5(15.6%)	0.380
Previous irradiation (yes)	1(4.5%)	2(25%)	1(3.3%)	1(4.4%)	0(0.0%)	0(0.0%)	0.022
Type implant							0.046
DTI	22(100.0%)	8(100.0%)	30(100.0%)	23(100.0%)	27(100.0%)	32(100.0%)	1.000
Tissue expander	0(0.0%)	0(0.0%)	0(0.0%)	0(0.0%)	0(0.0%)	0(0.0%)	
Type of reconstruction							<0.001
Pre-pectoral	6(27.3%)	4 (50.0%)	8(26.7%)	20(86.9%)	24(88.8%)	7(21.9%)	
Sub-pectoral	12(54.5%)	1 (12.5%)	9(30.0%)	1 (4.4%)	3(11.1%)	23(71.9%)	
Dual plane	4 (18.2%)	3 (37.5%)	13(43.3%)	2(8.7%)	0(0.0%)	2 (6.2%)	
Mesh (yes)	2 (9.1%)	3 (37.5%)	8(26.6%)	5(21.7%)	3(11.1%)	3 (9.5%)	0.180
T							0.085
TIS	1(4.5%)	1(12.5%)	4(13.3%)	1(4.3%)	5(18.5%)	2(6.1%)	
T0*	6(27.3%)	0(0.0%)	4(13.3%)	5(21.7%)	4(14.8%)	6(18.2%)	
T1	9(40.9%)	0(0.0%)	15(50.0%)	9(39.1%)	10(37.0%)	11(33.3%)	
T2	5(22.7%)	2(25.0%)	6(20.0%)	5(21.7%)	5(18.5%)	8(24.2%)	
T3	1(4.5%)	4(50.0%)	1(3.3%)	3(13.0%)	1(3.7%)	4(12.1%)	
T4	0(0.0%)	1(12.5%)	0(0.0%)	0(0.0%)	2(7.4%)	2(6.1%)	
N							0.727
N0	13(59.1%)	5(62.5%)	22(73.3%)	17(73.9%)	21(77.8%)	24(75.0%)	
N +	9(40.9%)	3(37.5%)	8(26.7%)	6(26.1%)	6(22.2%)	8(25.0%)	
Axillary surgery							0.251
SNLB	13(59.1%)	5(62.5%)	25(83.3%)	18(78.3%)	23(85.2%)	25(78.1%)	
ALND	9(40.9%)	3(37.5%)	5(16.6%)	5(18.51%)	4(14.8%)	7(21.9%)	
Mastectomy indications							0.001
Invasive carcinoma **	19 (86.4%)	6(75.0%)	26 (86.6%)	22(95.6%)	16 (59.2%)	30(93.7%)	
Pure DCIS	1 (4.5%)	1(12.5%)	4(13.3%)	1(4.4%)	5(18.5%)	2 (6.3%)	
Other***	2 (9.1%)	1(12.5%)	0(0.0%)	0(0.0%)	6(22.2%)	0(0.0%)	
Hospitalization (days)	2 [0–5]	2 [1–6]	2 [2, 3]	2 [0–3]	2 [2–4]	2 [0–6]	0.489
Surgical time (minutes)	211 [123–265]	122 [86–142]	207 [149–222]	211[104–201]	201 [137:296]	219 [131:395]	0.033
Follow- up (Months)	61[14–115]	50[23–119]	37[14–55]	40[19–73]	32[17–55]	47[15–61]	0.001
Adjuvant irradiation	1 (4.5%)	1(12.5%)	0(0.0%)	0(0.0%)	0(0.0%)	1 (3.1%)	0.244

*NAC* Nipple areola complex, *DTI* Direct to implant, *DCIS* Ductal carcinoma in situ

^*^Complete response after neoadjuvant chemotherapy

^**^Ductal or lobular carcinoma

^***^Mixed type, previous positive resection margin; hospitalization in days, surgical time in minutes

Reoperation was required in one patient (4.5%) in the radial incision group, two patients (7.4%) in the periareolar group, and three patients (9.4%) in the Wise-pattern group (*p* = 0.435). Although the difference was not statistically significant, a trend toward higher reoperation rates was noted in patients undergoing Wise-pattern incisions. Surgical time was significantly lower in patients subjected to skin excision, *p* = 0.033 (Table 3). Adjuvant breast irradiation was administered in one patient (4.5%) in the radial incision group, one patient (12.5%) in the skin excision group, and one patient (3.1%) in the Wise-pattern group. No cases were reported in the omega areolar and periareolar incision groups. The difference among groups was not statistically significant (*p* = 0.244) (Table 3).

The overall complication rate differed significantly among incision types (*p* = 0.001), as detailed in Table 4. Notably, no grade 3 or 4 complications were observed.

**Table 4:** Postoperative complications according to type of incision in patients undergoing nipple-sparing mastectomy for breast cancer

	Radial incision (*n* = 22)	Incision with skin excision (*n* = 8)	Inframammary fold incision (*n* = 30)	Omega areola incision (*n* = 23)	Periareolar incision (*n* = 27)	Wise-pattern incision (*n* = 32)	*p* value
Complications*	3(13.6%)	2(25.0%)	2(6.6%)	3(13%)	6(22.2%)	13(40.6%)	0.001
Grade 1	0(0.0%)	1(12.5%)	1(3.3%)	1(4.3%)	1(3.7%)	2(6.3%)	
Grade 2	2(9.1%)	1(12.5%)	1(3.3%)	2 (8.7%)	3(11.1%)	8(25.0%)	
Grade 3	1(4.5%)	0(0.0%)	0(0.0%)	0(0.0%)	2(7.4%)	3(9.4%)	
Reoperation	1(4.5%)	0(0.0%)	0(0.0%)	0(0.0%)	2(7.4%)	3(9.4%)	0.186
Temporary MSF ischemia	1(4.5%)	0(0.0%)	0(0.0%)	0(0.0%)	2(7.4%)	8(25.0%)	0.004
Delayed wound healing	1(4.5%)	0(0.0%)	0(0.0%)	0(0.0%)	0(0.0%)	3(9.4%)	0.188
NAC necrosis	1(4.5%)	0(0.0%)	0(0.0%)	1(4.3%)	1(3.7%)	4(12.5%)	0.213
Skin necrosis	1(4.5%)	0(0.0%)	0(0.0%)	0(0.0%)	0(0.0%)	4(12.5%)	0.054
Seroma	0(0.0%)	2(25.0%)	2(6.6%)	2 (8.7%)	3(11.1%)	1 (3.1%)	0.210
Hematoma	1(4.5%)	0(0.0%)	0(0.0%)	0(0.0%)	1(3.7%)	1 (3.1%)	0.802
Bleeding	0(0.0%)	0(0.0%)	0(0.0%)	0(0.0%)	1(3.7%)	0(0.0%)	0.555
Implant loss	1(4.5%)	0(0.0%)	0(0.0%)	0(0.0%)	1(3.7%)	3(9.4%)	0.435
Interval time to adjuvant treatment >60 (days)	1(4.5%)	1(12.5%)	0(0.0%)	2 (8.7%)	3(11.1%)	8(25.0%)	0.041

*MSF* Mastectomy skin flap, *NAC* Nipple areola complex

^*^Complications according to Clavien–Dindo^16^

Temporary MSF ischemia and skin necrosis occurred more frequently in the Wise-pattern incision group, reaching statistical significance (*p* = 0.004 and *p* = 0.036, respectively). Additional complications, their corresponding incidence rates, and statistical significance levels are summarized in Table 4.

The incidence of an interval to adjuvant treatment exceeding 60 days, indicating a potential oncologic delay, was significantly higher among patients undergoing Wise-pattern incisions (25%), followed by those with incisions involving skin excision (12.5%) and periareolar incisions (11.1%). This difference reached statistical significance (*p* = 0.041; Table 4).

### Multivariate analysis

Table 5 shows the ORs and relative 95% CI from multiple logistic regression analyses to identify risk factors for NAC necrosis. The Wise-pattern incision, smoking, and older age were identified as risk factors for NAC necrosis (Table 3). Differently, the inframammary fold incision was associated with a reduced risk of nipple necrosis (OR: 0.651 and *p* value 0.026;95%CI:0.241–0.986), while the Wise-pattern incision increased the risk (OR 3.611 and *p* value 0.038; 95%CI:1.567–8.239) (Table 3). Other variables evaluated in the multivariate logistic regression analysis, along with their respective ORs, 95% CIs, and *p* values are summarized in Table 3.

**Table 5:** Multivariate logistic regression analyses to identify risk factors for NAC necrosis

Multivariate			
	OR	95% CI	*p* value
Periareolar incision	2.211	0.564–5.733	0.141
Inframammary fold incision	0.656	0.241–0.986	0.026
Wise-pattern incision	3.611	1.567–8.239	0.038
Age (older)	1.617	1.047–1.658	0.019
Smokers	1.913	0.798–4.586	0.053
Diabetes	1.064	0.124–9.564	0.867
Pre-pectoral breast reconstruction	1.001	0.989–1.013	0.810

Differently, type of incision, age, and smoking were not found to be risk factors for implant loss. The relative OR and *p* values were: (OR: 0.388, *p* = 0.505, 95%CI: 0.221–1.139; OR: 0.262, *p* = 0.183, 95%CI: 0.191–2.868 and OR: 0.332, *p* = 0.380, 95%CI: 0.277–1.786).

## Discussion

A key topic of discussion when performing IBR after conserving mastectomy is whether preserving the NAC and the type of incision affects oncologic safety and the risk of NAC wound complications. Several studies have provided evidence supporting the oncological safety of NAC preservation [8–10]. While nipple preservation can increase the risk of NAC and skin necrosis, some studies have suggested that the choice of incision may also influence this risk [3, 12–16]. Although complications such as wound dehiscence, skin necrosis, and partial NAC necrosis often resolve spontaneously, they may delay adjuvant systemic therapy and potentially impair oncological outcomes [17]. Even though breast-conserving mastectomy, when oncologically feasible, should be the preferred approach, it is crucial to minimize the risk of complications. Our results demonstrated that the rates of overall skin necrosis, NAC necrosis, and delayed wound healing were higher in smokers and in patients undergoing conserving mastectomy with Wise-pattern and periareolar incisions compared to other incisions. According to our multivariate analysis, the IMF incision was associated with a reduced risk of NAC and skin necrosis.

These findings were confirmed in our sub-analysis, which included only patients undergoing NSM for oncologic indications. In this subgroup, patients who underwent NSM through an IMF incision experienced a lower incidence of postoperative complications. However, no statistically significant difference in NAC necrosis was observed. This may be attributed both to the thinner skin flaps typical of oncologic mastectomies, which could affect vascular supply, and to the relatively low absolute number of necrosis events reported in this population, despite the higher proportion observed in patients who underwent a Wise-pattern incision.

For NSM, there are several different types of incisions. These include radial incision (Fig. 1a), transverse with skin or NAC excision (Fig. 1b), inframammary fold incision (Fig. 1c), omega areola incision (Fig. 1d), periareolar incision (Fig. 1e), and Wise-pattern incision, used to reduce excess skin (Fig. 1f). The choice of incision is generally determined by the surgeon according to tumor location, breast volume, patient risk factors for NAC necrosis [3, 7–18]. At our institution, surgeons predominantly utilize the periareolar incision, with or without extension (omega incision). Differently, in younger patients, the IMF incision is preferred due to its hidden location and esthetic outcomes, despite being technically more challenging, especially in large or ptotic breasts [19].

In patients with large and ptotic breasts, a simple NSM may not provide satisfactory esthetic results due to excess skin. The Wise pattern, adapted for mastectomy, is a technique used to lift and reshape the breast by removing the excess tissue, making it a valuable option for breast reconstruction in these cases [20]. However, several studies have shown that Wise-pattern mastectomy is independently associated with an increased risk of wound-related complications, including NAC and skin necrosis [19–21]. This increased risk can be attributed to the greater extent of incision in Wise-pattern mastectomy, which can compromise the vascularization of the mastectomy skin flaps, which rely on the subdermal plexus [22]. However, in cases of breast ptosis, skin-reducing mastectomy is often necessary to achieve a good esthetic and symmetrical outcome.

In addition to the meticulous attention paid to vascular pedicles during Wise-pattern mastectomy surgery, different strategies are adopted to reduce the risk of complications in conserving mastectomy [20–25]. The two-stage NSM, described in 2012 by Jensen and colleagues, is a strategy to reduce the risk of necrosis [24]. The procedure involves first separating the central portion of the breast skin envelope, including the NAC, from the underlying breast tissue along the subcutaneous plane and then completing a second procedure to complete the mastectomy [24]. This is hypothesized to increase compensatory blood flow to the NAC from the surrounding skin prior to definitive NSM, reducing the postoperative necrosis [23]. Although this technique has demonstrated a reduction in the risk of necrosis events, it requires two surgical procedures, with a psychological impact on the patient and on the health costs. For these reasons, this strategy was not adopted in our institution. Alternatively, in cases of high necrosis risk in our center we prefer to position a slightly swollen tissue expander to stretch the skin without creating tension on the MSF, postponing the definitive implant reconstruction. Reducing MSF tension improves vascularization, therefore decreasing the potential necrosis risk, as reported in different studies [20, 21]. This strategy was adopted in about 20% of cases in our analysis to reduce the risk of NAC or skin necrosis in patients undergoing either NSM or Wise-pattern mastectomy. A significant reduction in terms of postoperative complications in patients undergoing temporary breast reconstruction has been reported by Escandón et al. [20].

A new, more modern technique is the assessment of MSF viability using indocyanine green fluorescence angiography [25, 26]. When tissue perfusion is compromised, reconstruction can be avoided by placing a tissue expander and reducing tension on the flaps. This postoperative complication can be prevented by intraoperative assessment of mastectomy skin flap viability and intervention when tissue perfusion is compromised. Indocyanine green fluorescence angiography is presumed to be a better predictor of MSFN compared to clinical evaluation alone.

The controversy over which incision type in conserving mastectomy is superior still remains debated. Frey and colleagues analyzed the necrosis complications in patients undergoing conserving mastectomy, reporting a higher incidence in Wise-pattern and periareolar incision [27]. In their analysis of 1207 NSM, Frey and colleagues reported that vertical radial incisions and IMF incisions had lower complication rates compared to the majority of other incision types [27]. Park et al. in 2020 reported that NAC necrosis occurred more frequently with periareolar incisions than with IMF incisions [12]. Consistent with these findings and our analysis, Daar et al. in a meta-analysis including more than eight thousand NSM, reported a significantly higher incidence of NAC necrosis when mastectomy was performed with a periareolar incision [28].

Contrary to these results, Paek et al. reported no significant difference between periareolar and other incisions in 34 patients undergoing NSM [29].

Based on these findings and several studies, IMF incisions appears to be the safest in terms of complications and offer the significant advantage of minimizing postoperative scar burden. In addition, breast shape and contour are less likely to be affected by scar maturation, fibrosis, and contraction when compared to other incision types, where scars are located on the breast mound. Surgical access for NSM with IMF incisions can be challenging and is best suited for patients with small- to moderate-sized breasts. In patients with large breasts, an IMF incision could be disadvantageous, as it is more difficult to dissect the upper pole [30]. In our analysis, we considered only patients undergoing implant-based reconstruction, excluding those patients with autologous reconstruction. This type of incision can limit access to recipient vessels during autologous flap reconstruction.

Although ischemic complication seems to be correlated with incision types, other complications as seroma, hematoma, bleeding, infection, and implant loss may not be directly related with the incision type. Similar results were reported by Soo Hyun Woo analysis [3].

In our analysis, we observed a statistically significant difference in the distribution of patients with prior breast irradiation. None of the patients with previous radiotherapy underwent a Wise-pattern incision, likely reflecting a cautious approach due to the associated higher risk of complications. Although prior irradiation in patients undergoing NSM is commonly associated with increased rates of postoperative complications, this correlation was not confirmed in our cohort [31]. A possible explanation is that, in our institution, patients with a history of radiotherapy are preferentially selected for autologous breast reconstruction rather than implant-based approaches. Consequently, the proportion of irradiated patients in our series is lower than what is typically reported in the literature [31, 32].

Interestingly, our findings are in line with those of Alperovich et al. who, in a retrospective study, reported no significant impact of prior radiotherapy on postoperative complication rates following NSM [32]. Similar results were recently confirmed by King et al. in 2023[33].

Delay in adjuvant treatment was significantly higher in patients subjected to Wise-pattern incision. Even when complications resolve without the need for reoperation, they can still delay the initiation of adjuvant treatment [34]. A recent study including 282 patients undergoing oncoplastic breast reduction surgery, not skin-reducing mastectomies, reported delays in adjuvant treatment [35]. Moen et al. concluded that reduction surgery should be avoided in patients with more aggressive tumors who are potential candidates for adjuvant therapy [35]. In the same way, we strongly believe that the Wise-pattern incision should be avoided in patients with aggressive disease who are potential candidates for adjuvant chemotherapy and who are at high risk for complications (e.g., obesity, smokers, type II diabetes, severe ptosis). In these patients, a two-stage mastectomy or alternative surgical strategies may be indicated to avoid such delays [24]. This study has few limitations: first, the retrospective nature of the study, the single-center experience, and the small sample size of analyzed patients. Moreover, the choice of incision type could be influenced by patients’ risk factors, indications for NSM, as well as breast volume and shape, potentially leading to a significant selection bias. Second, the distribution of the cohort groups was not obtained. Patients undergoing IMF incisions were relatively young and had lower BMI, as our breast surgeons primarily performed the IMF incisions in selected young breast cancer patients, which could have impacted the risk of complications. Another limitation of the study is the difference in follow-up duration among the groups according to the type of incision performed. Although all included patients had a minimum follow-up of 12 months, with a median follow-up of at least 30 months, this difference could represent a potential bias, particularly regarding the complication of implant loss. Despite these limitations, we believe that the factor analysis was conducted relatively well in this study compared to the clinical results of other recent retrospective studies. Furthermore, it provides a basis for prospective randomized studies aimed at identifying the best incision type for mastectomy.

## Conclusion

Wise-pattern incision and periareolar incision in NSM are associated with a higher incidence of complications, including NAC necrosis, especially in patients with other risk factors as DMII and smoking. However, the type of incision does not appear to affect reconstruction failure. When feasible, IMF should be the preferred incision due to its low risk of complications and good esthetic results. In cases of breast ptosis, the Wise pattern remains a valid option, but special attention should be paid to reduce the risk of necrosis through all possible strategies.

We believe that there is no single optimal incision, but rather that the choice should be customized based on tumor location, biology and extent, breast volume, degree of ptosis, type of reconstruction, and risk factors.

## Data Availability

The data that support the findings of this study are available from the corresponding author after formal request.

## References

[1] Veronesi U, Cascinelli N, Mariani L, Greco M, Saccozzi R, Luini A, Aguilar M, Marubini E (2002) Twenty-year follow-up of a randomized study comparing breast-conserving surgery with radical mastectomy for early breast cancer. N Engl J Med 347(17):1227–1232. 10.1056/NEJMoa020989 12393819

[2] Prasad K, Zhou R, Zhou R, Schuessler D, Ostrikov KK, Bazaka K (2019) Cosmetic reconstruction in breast cancer patients: opportunities for nanocomposite materials. Acta Biomater 86:41–65. 10.1016/j.actbio.2018.12.024 30576863

[3] Woo SH, Choi JM, Eom JS, Kim EK, Han HH (2022) Outcome analysis depending on the different types of incision following immediate breast reconstruction. Breast J 2022:7339856. 10.1155/2022/7339856 PMC9187267 35711891

[4] Wu ZY, Kim HJ, Lee J, Chung IY, Kim J, Lee SB, Son BH, Han J, Han HH, Eom JS, Kim SB, Jung KH, Gong G, Kim HH, Ahn SH, Ko B (2021) Oncologic safety of nipple-sparing mastectomy in patients with breast cancer and tumor-to-nipple distance ≤ 1 cm: a matched cohort study. Ann Surg Oncol 28(8):4284–4291. 10.1245/s10434-020-09427-0 33423119

[5] Mota BS, Riera R, Ricci MD, Barrett J, de Castria TB, Atallah ÁN, Bevilacqua JL (2016) Nipple- and areola-sparing mastectomy for the treatment of breast cancer. Cochrane Database Syst Rev 11(11):CD008932. 10.1002/14651858.CD008932.pub3 PMC5868722 27898991

[6] van Deventer PV, van Deventer PV (2004) The blood supply to the nipple-areola complex of the human mammary gland. Aesthet Plast Surg 28(6):393–398. 10.1007/s00266-003-7113-9 15633023

[7] Peled AW, Foster RD, Ligh C, Esserman LJ, Fowble B, Sbitany H (2014) Impact of total skin-sparing mastectomy incision type on reconstructive complications following radiation therapy. Plast Reconstr Surg 134(2):169–175. 10.1097/PRS.0000000000000386 24732652

[8] Sacchini V, Pinotti JA, Barros AC, Luini A, Pluchinotta A, Pinotti M, Boratto MG, Ricci MD, Ruiz CA, Nisida AC, Veronesi P, Petit J, Arnone P, Bassi F, Disa JJ, Garcia-Etienne CA, Borgen PI (2006) Nipple-sparing mastectomy for breast cancer and risk reduction: oncologic or technical problem? J Am Coll Surg 203(5):704–714. 10.1016/j.jamcollsurg.2006.07.015 17084333

[9] Garwood ER, Moore D, Ewing C, Hwang ES, Alvarado M, Foster RD, Esserman LJ (2009) Total skin-sparing mastectomy: complications and local recurrence rates in 2 cohorts of patients. Ann Surg 249(1):26–32. 10.1097/SLA.0b013e31818e41a7 19106672

[10] Algaithy ZK, Petit JY, Lohsiriwat V, Maisonneuve P, Rey PC, Baros N, Lai H, Mulas P, Barbalho DM, Veronesi P, Rietjens M (2012) Nipple sparing mastectomy: can we predict the factors predisposing to necrosis? Eur J Surg Oncol 38(2):125–129. 10.1016/j.ejso.2011.10.007 22056645

[11] Galimberti V, Vicini E, Corso G, Morigi C, Fontana S, Sacchini V, Veronesi P (2017) Nipple-sparing and skin-sparing mastectomy: review of aims, oncological safety and contraindications. Breast 34(1):S82–S84. 10.1016/j.breast.2017.06.034 PMC5837802 28673535

[12] Park S, Yoon C, Bae SJ, Cha C, Kim D, Lee J, Ahn SG, Roh TS, Kim YS, Jeong J (2020) Comparison of complications according to incision types in nipple-sparing mastectomy and immediate reconstruction. Breast 53:85–91. 10.1016/j.breast.2020.06.009 PMC7375566 32653836

[13] Ahn SJ, Woo TY, Lee DW, Lew DH, Song SY (2018) Nipple-areolar complex ischemia and necrosis in nipple-sparing mastectomy. Eur J Surg Oncol 44(8):1170–1176. 10.1016/j.ejso.2018.05.006 29859649

[14] De Vita R, Zoccali G, Buccheri EM, Costantini M, Botti C, Pozzi M (2017) Outcome evaluation after 2023 nipple-sparing mastectomies: our experience. Plast Reconstr Surg 139(2):335e–347e. 10.1097/PRS.0000000000003027 28121852

[15] Santanelli F, Paolini G, Campanale A, Longo B, Amanti C (2010) The, “Type V” skin-sparing mastectomy for upper quadrant skin resections. Ann Plast Surg 65(2):135–139. 10.1097/SAP.0b013e3181c60f41 20657195

[16] Clavien PA, Barkun J, de Oliveira ML et al (2009) The clavien-dindo classification of surgical complications: five-year experience. Ann Surg 250(2):187–196. 10.1097/SLA.0b013e3181b13ca2 19638912

[17] Vanni G, Pellicciaro M, Buonomo OC (2024) Axillary lymph node dissection in breast cancer patients: obsolete or still necessary? The Lancet Regional Health 47:101107. 10.1016/j.lanepe.2024.101107 PMC11670684 39726723

[18] Dayicioglu D, Tugertimur B, Zemina K et al (2016) Vertical mastectomy incision in implant breast reconstruction after skin sparing mastectomy: advantages and outcomes. Ann Plast Surg 76(4):S290–S294 10.1097/SAP.0000000000000676 27187251

[19] Cavalcante FP, Lima TO, Alcantara R, Cardoso A, Ulisses F, Novita G, Zerwes F, Millen E (2023) Inframammary versus periareolar incision: a comparison of early complications in nipple-sparing mastectomy. Plast Reconstr Surg 11(11):e5367. 10.1097/GOX.0000000000005367 PMC10624459 37928633

[20] Escandón JM, Butterfield JA, Christiano JG, Gooch JC, Olzinski AT, Prieto PA, Skinner KA, Langstein HN, Manrique OJ (2023) Wise pattern versus transverse pattern mastectomy in two-stage implant-based breast reconstruction: a propensity score-matched analysis. Plast Reconstr Surg 152(4S):69S-80S. 10.1097/PRS.0000000000010703 37220238

[21] Corban J, Shash H, Safran T, Sheppard-Jones N, Fouda-Neel O (2017) A systematic review of complications associated with direct implants vs. tissue expanders following Wise pattern skin-sparing mastectomy. J Plast Reconstr Aesthet Surg 70(9):1191–1199. 10.1016/j.bjps.2017.02.028 28410984

[22] Ponzone R, Maggiorotto F, Carabalona S, Rivolin A, Pisacane A, Kubatzki F, Renditore S, Carlucci S, Sgandurra P, Marocco F, Magistris A, Regge D, Martincich L (2015) MRI and intraoperative pathology to predict nipple-areola complex (NAC) involvement in patients undergoing NAC-sparing mastectomy. Eur J Cancer 51(14):1882–1889. 10.1016/j.ejca.2015.07.001 26210374

[23] Ju T, Chandler J, Momeni A, Gurtner G, Tsai J, Nguyen D, Wapnir I (2021) Two-stage versus one-stage nipple-sparing mastectomy: timing of surgery prevents nipple loss. Ann Surg Oncol 28(10):5707–5715. 10.1245/s10434-021-10456-6 34291379

[24] Jensen JA, Lin JH, Kapoor N (2012) Surgical delay of the nipple-areolar complex: a powerful technique to maximize nipple viability following nipple-sparing mastectomy. Ann Surg Oncol 19(10):3171–3176. 10.1245/s10434-012-2528-7 22829005

[25] Hulst RR, Qiu SS (2020) Indocyanine green angiography for preventing postoperative mastectomy skin flap necrosis in immediate breast reconstruction. Cochrane Database Syst Rev 4(4):CD013280. 10.1002/14651858.CD013280.pub2. PMID: 32320056; PMCID: PMC7175780. PMC7175780 32320056

[26] Pagliara D, Serra PL, Pili N, Giardino FR, Grieco F, Schiavone L, Lattanzi M, Rubino C, Ribuffo D, De Santis G, Salgarello M, Nahabedian MY, Rancati A (2024) Prediction of mastectomy skin flap necrosis with indocyanine green angiography and thermography: a retrospective comparative study. Clin Breast Cancer 24(8):755–762. 10.1016/j.clbc.2024.08.024 39341758

[27] Frey JD, Salibian AA, Levine JP, Karp NS, Choi M (2018) Incision choices in nipple-sparing mastectomy: a comparative analysis of outcomes and evolution of a clinical algorithm. Plast Reconstr Surg 142(6):826e–835e. 10.1097/PRS.0000000000004969 30204677

[28] Daar DA, Abdou SA, Rosario L, Rifkin WJ, Santos PJ, Wirth GA, Lane KT (2019) Is there a preferred incision location for Nipple-sparing mastectomy? A systematic review and meta-analysis. Plast Reconstr Surg 143(5):906e–919e. 10.1097/PRS.0000000000005502 30789474

[29] Paek SH, Hong SE, Woo KJ, Woo J, Lim W (2020) Nipple-sparing mastectomy through periareolar incision with immediate reconstruction. Ann Surg Treat Res 98(2):57–61. 10.4174/astr.2020.98.2.57 PMC7002881 32051813

[30] Sisco M, Yao KA (2016) Nipple-sparing mastectomy: a contemporary perspective. J Surg Oncol 113(8):883–890. 10.1002/jso.24209 26931641

[31] Tang R, Coopey SB, Colwell AS et al (2015) Nipple-sparing mastectomy in irradiated breasts: selecting patients to minimize complications. Ann Surg Oncol 22(10):3331–3337. 10.1245/s10434-015-4669 26202557

[32] King CA, Bartholomew AJ, Dabic S et al (2023) Nipple-sparing mastectomy with immediate reconstruction after breast-conserving therapy and radiation: complications and oncologic safety. Ann Plast Surg 91(6):709–714. 10.1097/SAP.0000000000003692 37830503

[33] Alperovich M, Choi M, Frey JD et al (2014) Nipple-sparing mastectomy in patients with prior breast irradiation: are patients at higher risk for reconstructive complications? Plast Reconstr Surg 134(2):202e–206e. 10.1097/PRS.0000000000000321 25068341

[34] Eck DL, McLaughlin SA, Terkonda SP, Rawal B, Perdikis G (2015) Effects of immediate reconstruction on adjuvant chemotherapy in breast cancer patients. Ann Plast Surg 74(Suppl 4):S201–S203. 10.1097/SAP.0000000000000446 25664417

[35] Moen M, Holton T, Phung A, et al (2024) Complications after oncoplastic breast reduction and impact on time to adjuvant therapy. Plast Reconstr Surg Glob Open 12(7):e6010. 10.1097/GOX.0000000000006010 PMC11288615 39081812

